# Invasive Fungal Rhinosinusitis versus Bacterial Rhinosinusitis with Orbital Complications: A Case-Control Study

**DOI:** 10.1155/2013/453297

**Published:** 2013-11-03

**Authors:** Patorn Piromchai, Sanguansak Thanaviratananich

**Affiliations:** Department of Otorhinolaryngology, Faculty of Medicine, Khon Kaen University, Khon Kaen 40002, Thailand

## Abstract

*Background*. Invasive fungal rhinosinusitis with orbital complications (IFSwOC) is a life-threatening condition. The incidence of mortality has been reported to be up to 80 percent. This study was conducted to determine the risk factors, presentations, clinical, and imaging findings that could help to manage this condition promptly. *Methods*. We conducted a case-control study of 100 patients suffering from rhinosinusitis with orbital complications. The risk factors, clinical presentations, radiological findings, medical and surgical managements, durations of hospital stay, and mortality rate data were collected. *Results*. Sixty-five patients were diagnosed with IFSwOC, while the other thirty-five patients composed the control group. The most important risk factor for IFSwOC was diabetes mellitus. Visual loss and diplopia were the significant symptom predictors. The significant clinical predictors were nasal crust, oculomotor nerve, and optic nerve involvement. The CT findings of IFSwOC were sinus wall erosion and hyperdensity lesions. The mortality rate was 25.71 percent in the IFSwOC group and 3.17 percent in the control group. *Conclusions*. Invasive fungal rhinosinusitis with orbital complications is symptomatic of a high mortality rate. The awareness of a patient's risk factors, the presenting symptoms, signs of fungal invasion, and aggressive management will determine the success of any treatment procedures.

## 1. Background

Invasive fungal rhinosinusitis with orbital complications (IFSwOC) is a challenging condition that is commonly seen in immunocompromised patients. The diagnosis of invasive fungal rhinosinusitis requires histopathologic evidence of fungi invading nasal tissue; hyphal formations within the mucosa, the submucosa, the blood vessels or the bones present around the sinus area [1, 2]. The invasion of the fungi usually spreads beyond the sinus cavity into the orbit and the intracranial space. The orbital complications include preseptal cellulitis, orbital cellulitis, subperiosteal abscess, and orbital abscess. Intracranial complications include epidural or subdural abscess, brain abscess, meningitis, encephalitis, and cavernous sinus thrombosis.

The incidence of morbidity and mortality among patients with complications arising from rhinosinusitis has been reported to range from 5 to 40 percent [3, 4]. The incidence was significantly higher when fungal invasion was detected, ranging from 20 to 80 percent [5].

The diagnosis of invasive fungal rhinosinusitis with orbital complications is usually delayed because the detection of fungal cultures or pathological results require a few days to a few weeks to be complete. Therefore, the presentations and the clinical findings obtained from IFSwOC patients are an important determinant. An early detection of fungal invasion will allow for better management and a better prognosis for the patient.

The purpose of this study was to determine the risk factors, presentations, clinical, and imaging findings that could help increase the awareness of symptoms derived from invasive fungal rhinosinusitis with orbital complications that require urgent intervention. We also review the treatment and outcome in patients demonstrating orbital complications derived from rhinosinusitis.

## 2. Methods

### 2.1. Study Design and Setting

We conducted a case-control study of invasive fungal rhinosinusitis with orbital complications between January 1997 and June 2008 at Srinagarind Hospital, Khon Kaen University. The hospital is the largest tertiary hospital in the north east region of Thailand. Most patients suffering from rhinosinusitis with orbital complications were referred to our hospital.

### 2.2. Case and Control Definition

Rhinosinusitis is defined as an inflammation of the nose and the paranasal sinuses and is characterised by two or more symptoms, which should be either a nasal blockage/obstruction/congestion or a nasal discharge (anterior/posterior nasal drip) ± facial pain/pressure ± reduction or loss of smell. These symptoms should be supported by a demonstrable disease that includes any of the following observations: endoscopic signs of nasal polyps, mucopurulent discharge primarily from the middle meatus, oedema/mucosal obstruction primarily in the middle meatus, or CT/mucosal changes within the ostiomeatal complex and/or sinuses [6, 7].

The orbital complications of rhinosinusitis were classified according to Chandler's classification [8] into the following.

- Preseptal cellulitis.
- Orbital cellulitis.
- Subperiosteal abscess.
- Orbital abscess.
- Cavernous sinus thrombosis.

The patients investigated were those suffering from invasive fungal rhinosinusitis with orbital complications. Fungal rhinosinusitis was defined as the histopathological evidence indicating the invasion of fungi into the nasal mucosa, the sinus mucosa, or deeper tissues. The control group that was investigated comprised patients suffering from bacterial rhinosinusitis with orbital complications. Bacterial rhinosinusitis with orbital complications was defined as the absence of histopathological evidence indicating the invasion of fungi into the nasal mucosa, the sinus mucosa, or deeper tissues.

### 2.3. Clinical and Pathological Evaluation

We collected the data from our rhinosinusitis registry, OPD cards, and admission records. Clinical symptoms included headaches, visual loss, facial pain, diplopia, stuffiness, fevers, postnasal drips, watery rhinorrhea, purulent rhinorrhea, maxillary toothaches, coughs, hyposmia, and changes in consciousness.

The clinical signs that were obtained included nasal endoscopies and orbital examinations. The presence of comorbidities, medical and surgical management, duration of hospital stay, and mortality were noted in a standardised checklist.

### 2.4. Radiological Evaluation

We performed a contrast-enhanced CT scan of the paranasal sinuses with axial, coronal, and sagittal cuts in all cases of suspected abscesses or cavernous sinus thrombosis. The CT scan was also performed in some cases where preseptal and orbital cellulitis were suspected. The radiological findings that were collected including air-fluid level, opacity, mucosal thickening, sinus wall erosion, hyperdensity lesions, sinus involvement, cavernous sinus involvement, intraorbital involvement, and laterality.

### 2.5. Statistical Analysis

The categorical variables were presented in the form of frequencies and percentages. The association between categorical variables was assessed using the chi-square test. The continuous variables were presented in the form of means. Factors affecting the outcome were assessed using a Student's *t*-test analysis, with a *P* value of less than 0.05 being considered statistically significant. The odds ratio adjusted by orbital complications severity was calculated to compare the risk factors between the two groups at a 95% confidence interval (CI). All statistical analyses were performed using the Statistical Package for the Social Sciences (SPSS Inc., Chicago, IL) software program, version 20.0.

### 2.6. Ethics

Approval was sought from the Khon Kaen University Ethics Committee for Human Research before initiating the study. As this study was a retrospective study, the need for an informed consent was waived by the ethics review board.

## 3. Results 

One hundred patients were included in this study. Sixty-five patients were diagnosed to be suffering from invasive fungal rhinosinusitis with orbital complications, while thirty-five patients were diagnosed to be suffering from bacterial rhinosinusitis with orbital complications. There were 53 males and 47 females. The average age of the patients was 46.63 years and ranged from 1 to 82.

In the IFSwOC group, the most common orbital complications were cavernous sinus thrombosis (42.86%) and subperiosteal abscess (40%). In the control group, the most common orbital complications were subperiosteal abscess (43.08%) and preseptal cellulitis (27.69%). The most important underlying risk factor for IFSwOC was diabetes mellitus (80%) (Table 1). 

The most common symptoms presented by both groups were orbital pain (47.4%), followed by fever (43.8%) and orbital swelling (45.6%). Visual loss (adjusted OR 3.12, 95% CI 1.06–9.22) and diplopia (adjusted OR 3.03, 95% CI 1.08–8.52) were the significant symptom predictors for IFSwOC (Table 2). 

The significant clinical predictors for the IFSwOC group were nasal crust (adjusted OR 77.7, 95% CI 81.95–3095.00), occulomotor nerve involvement (adjusted OR 15.11, 95% CI 2.16–644.26), and optic nerve involvement (adjusted OR 3.77, 95% CI 1.50–9.50) (Table 3). 

The significant CT findings in the IFSwOC group included sinus wall erosions and hyperdensity lesions (adjusted OR 4.61, 95% CI 1.20–17.82). The common finding between both groups was that they commonly had more than two sinus cavities that were involved (Table 4). 

All patients in the IFSwOC group underwent endoscopic debridement or external approach and received amphotericin B. The average hospital stay for the IFSwOC and the control groups were 34.97 ± 29.32 and 14 ± 8.48 days, respectively. The mortality rate was 25.71 percent in IFSwOC group and 3.17 percent in the control group.

## 4. Discussion 

An increase in the prevalence of invasive fungal invasion in rhinosinusitis is thought to be secondary to the increasing numbers of immunocompromised patients [9–11]. Medical advancements have prolonged the survival of immunocompromised patients, which has, in turn, increased the proportion of the population at risk for developing invasive fungal rhinosinusitis [12]. Survival is dependent on the early detection of the disease, followed by aggressive surgical and medical management [10].

We found that diabetes mellitus was the most common underlying risk factor for invasive fungal rhinosinusitis with orbital complications (80 percent). Poorly controlled diabetics face the greatest risk of infection; however, any immunocompromised individual may also be infected [13].

The invasive fungal rhinosinusitis group had a more severe form of orbital complications such as cavernous sinus thrombosis (42.86 percent). The symptoms have usually been subtle and initially hard to diagnose because of the nature of fungal infections [14]. On the other hand, bacterial rhinosinusitis tends to be less severe, but could be present at any stage of the orbital complications.

The persistent symptoms that could alert us that a patient was suffering from invasive fungal rhinosinusitis with orbital complications were visual loss and diplopia. There were no specific nasal symptoms that could differentiate fungal from bacterial infections. Both nasal and orbital examinations were important. We found that nasal crust and cranial nerve involvement were predictors of fungal infection. Conventional anterior rhinoscopy and posterior rhinoscopy may not be adequate to visualise some parts of the anatomy such as the middle meatus. We encourage all patients to complete an endoscopic nasal examination. The CT scanning of paranasal sinus is an essential tool for diagnosing invasive fungal rhinosinusitis with orbital complications. Sinus wall erosions and hyperdensity lesions were found to be predictors of fungal invasion.

The treatment of invasive fungal rhinosinusitis with orbital complications included a combination of antifungal antibiotics with aggressive surgical debridement [15–19]. A dosage of amphotericin B at 2 grams or higher was an important adjunct in the treatment of invasive fungal rhinosinusitis.

This study has the limitation of being a retrospective case-control study. Limitations arise from sample selection and data collection. This analysis cannot reflect every aspect of invasive fungal rhinosinusitis with orbital complications.

## 5. Conclusions 

Invasive fungal rhinosinusitis with orbital complications still present a high mortality rate. The awareness of the patient's risk factors, presenting symptoms, signs of fungal invasion, and aggressive management will determine the success of treatment.

## Figures and Tables

**Table 1:** Demographic data.

	Invasive fungal rhinosinusitis with orbital complications (*n* = 35)	Bacterial rhinosinusitis with orbital complications (*n* = 65)
Sex (male : female)	16 : 19	37 : 28
Age (mean ± SD)	53.80 ± 12.71	42.77 ± 22.07
Underlying diseases (%)		
DM	28 (80)	14 (21.54)
Hematologic diseases	1 (2.86)	12 (18.46)
Hypertension	6 (17.14)	8 (12.31)
Renal failure	2 (5.71)	8 (12.31)
SLE	0	2 (3.08)
Orbital complications (%)		
Preseptal cellulitis	2 (5.71)	18 (27.69)
Orbital cellulitis	0	5 (7.69)
Subperiosteal abscess	14 (40)	28 (43.08)
Orbital abscess	4 (11.43)	2 (3.08)
Cavernous sinus thrombosis	15 (42.86)	12 (18.46)

**Table 2:** Symptoms of invasive fungal rhinosinusitis versus bacterial rhinosinusitis with orbital complications adjusted by severity of orbital complications.

	Invasive fungal rhinosinusitis with orbital complications (*n* = 35)	Bacterial rhinosinusitis with orbital complications (*n* = 65)	Adjusted odds ratio (95% CI)	*P* value
Visual loss	27	27	3.12 (1.06–9.22)	0.03
Diplopia	12	6	3.03 (1.08–8.52)	0.04
Headaches	23	27	1.96 (0.67–5.64)	0.21
Postnasal drips	2	4	1.78 (0.18–18.01)	0.61
Watery rhinorrhea	8	12	1.63 (0.41–3.98)	0.60
Facial pains	14	20	1.49 (0.59–3.77)	0.40
Purulent rhinorrhea	6	9	1.20 (0.53–5.00)	0.39
Coughs	3	7	1.10 (0.20–6.07)	0.91
Stuffiness	5	13	0.94 (0.31–2.89)	0.91
Fevers	11	28	0.82 (0.31–2.13)	0.68
Changes in consciousness	1	4	0.40 (0.11–1.41)	0.15
Toothaches	2	10	0.38 (0.07–2.30)	0.28

**Table 3:** Clinical signs of invasive fungal rhinosinusitis versus bacterial rhinosinusitis with orbital complications adjusted by severity of orbital complications.

	Invasive fungal rhinosinusitis with orbital complications (*n* = 35)	Bacterial rhinosinusitis with orbital complications (*n* = 65)	Adjusted odds ratio (95% CI)	*P* value
Nasal crust	14	1	77.78 (1.95–3095.00)	<0.001
Oculomotor nerve involvement	34	45	15.11 (2.16–644.26)	0.001
Trochlear nerve involvement	33	43	5.70 (0.52–62.57)	0.10
Optic nerve involvement	22	17	3.77 (1.50–9.50)	0.002
Abducens nerve involvement	33	45	3.70 (0.40–34.57)	0.22
Facial swelling	10	8	2.37 (0.82–6.80)	0.10
Trigeminal nerve involvement	16	13	1.62 (0.77–3.42)	0.20
Pus in middle meatus	16	27	1.36 (0.57–3.25)	0.49
Swelling of middle meatus	28	31	1.29 (0.55–3.04)	0.56
Secretion in nasopharynx	4	10	0.56 (0.18–1.73)	0.31
Proptosis	27	45	0.53 (0.16–1.79)	0.30
Periorbital swelling	20	58	0.32 (0.12–0.83)	0.01
Chemosis	27	53	0.26 (0.06–1.18)	0.06

**Table 4:** Computed tomography findings of invasive fungal rhinosinusitis versus bacterial rhinosinusitis with orbital complications adjusted by severity of orbital complications.

	Invasive fungal rhinosinusitis with orbital complications (*n* = 35)	Bacterial rhinosinusitis with orbital complications (*n* = 65)	Adjusted odds ratio (95% CI)	*P* value
Sinus wall erosion	10	0	—	—
Hyperdensity lesions	6	4	4.61 (1.20–17.82)	0.01
Pan sinus involvement	8	16	0.89 (0.31–2.61)	0.85
Maxillary sinus involvement only	2	13	0.43 (0.08–2.38)	0.32
Sphenoid sinus involvement only	7	5	1.42 (0.40–5.06)	0.60
Two sinus or more involvement	26	44	1.41 (0.52–3.90)	0.49
